# Shengu granules ameliorate ovariectomy-induced osteoporosis by the gut-bone-immune axis

**DOI:** 10.3389/fmicb.2024.1320500

**Published:** 2024-03-08

**Authors:** Xiao cong Chen, Wei ju Li, Jia ying Zeng, Yun peng Dong, Jian ming Qiu, Bing Zhang, Dong yang Wang, Jun Liu, Zhao hui Lyu

**Affiliations:** ^1^Guangzhou University of Chinese Medicine, Guangzhou, China; ^2^The Fifth Clinical College of Guangzhou University of Chinese Medicine, Guangzhou, China; ^3^Guangdong Provincial Second Hospital of Traditional Chinese Medicine, Guangdong Provincial Engineering Technology Research Institute of Traditional Chinese Medicine, Guangzhou, China; ^4^The Research Team on Bone and Joint Degeneration and Injury, Guangdong Provincial Academy of Traditional Chinese Medicine, Guangzhou, China

**Keywords:** Gut microbiota, Shengu granules, PMOP, SCFAs, Inflammatory factors, Traditional Chinese medicine, The gut-bone-immune axis

## Abstract

**Introduction:**

Postmenopausal osteoporosis (PMOP) is a common chronic disease, and the loss of bone density and bone strength after menopause are its main symptoms. Effective treatments for PMOP are still uncertain, but Chinese medicine has some advantages in slowing down bone loss. Shengu granules are often used clinically to treat PMOP. It has been shown to be an effective prescription for the treatment of PMOP, and there is evidence that gut flora may play an important role. However, whether Shengu granules attenuate PMOP by modulating gut flora and related mechanisms remains unclear.

**Methods:**

In this study, we mainly examined the bone strength of the femur, the structure of the intestinal microbiota, SCFAs in the feces and the level of FOXP3 cells in the colon. To further learn about the inflammation response, the condition of the mucosa and the level of cytokines in the serum also included in the testing. In addition, to get the information of the protein expression, the protein expression of OPG and RANKL in the femur and the protein expression of ZO-1 and Occludin in the colon were taken into account.

**Results:**

The osteoporosis was significantly improved in the SG group compared with the OVX group, and the diversity of intestinal flora, the secretion level of SCFAs and the expression level of FOXP3 were significantly increased compared with the OVX group. In terms of inflammatory indicators, the intestinal inflammation scores of the SG group was significantly lower than those in the OVX group. Additionally, the serum expression levels of IL-10 and TGF-β in the SG group were significantly increased compared with the OVX group, and the expression levels of IL-17 and TNF-α were significantly decreased compared with the OVX group. In terms of protein expression, the expression levels of ZO-1, Occluding and OPG were significantly increased in the SG group compared with the OVX group, and the expression level of RANKL was significantly decreased compared with the OVX group.

**Discussion:**

Shengu granules treatment can improve the imbalance of intestinal flora, increase the secretion of SCFAs and the expression of FOXP3, which reduces the inflammatory response and repairs the intestinal barrier, as well as regulates the expression of OPG/RANKL signaling axis. Overall, Shengu granules ameliorate ovariectomy-induced osteoporosis by the gut-bone-immune axis.

## Graphical abstract

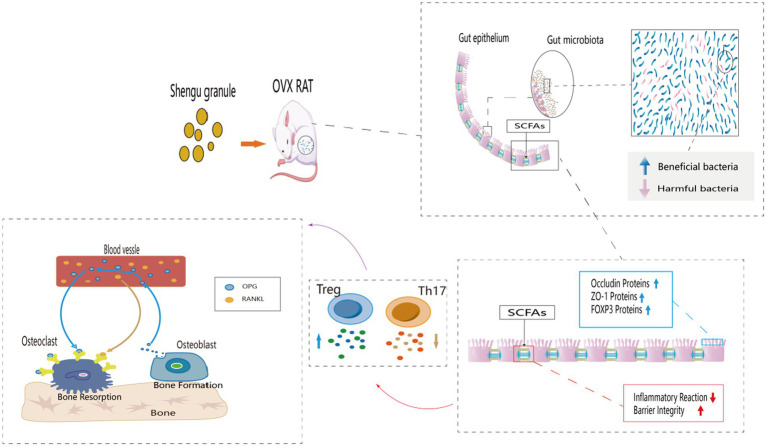

## 1 Introduction

Postmenopausal osteoporosis (PMOP) is a common systemic metabolic bone disease in elderly women, characterized by the decline of ovarian function and insufficient secretion of estrogen (E2) after menopause, resulting in bone loss and bone microstructural deterioration. With aging and a decline in E2 secretion, postmenopausal women lose 15% to 20% more bone than men (Huidrom et al., 2021). Nearly 30%–50% of menopausal women will bring about osteoporosis, and its incidence will increase with the prolongation of menopausal time (Arceo-Mendoza and Camacho, 2021). Osteoporosis can also be called a “silent killer” because patients often have no discomfort or symptoms until serious consequences like fractures happen. The rapid decline in estrogen in postmenopausal older women has led to a rapid increase in the incidence of PMOP, which has become an significant global public health issue (Clynes et al., 2020). Currently PMOP is mostly treated and prevented with medication (Johnston and Dagar, 2020). Current mainstream drugs include dual-acting agents, bone resorption inhibitors, osteogenesis promoters, and medications with different mechanisms (Ayariga et al., 2022). Prolonged use of these medications can easily result in a wide range of negative effects (Johnston and Dagar, 2020), therefore there is an urgent need for innovative preventative and therapeutic approaches. In recent years, the “gut-bone” axis has been put out as a novel approach for the prevention and treatment of PMOP. Hormones, immunity, nutrient metabolism, gut flora metabolites, and intestinal permeability have been recognized as potential mechanisms linking gut flora and PMOP (Xu et al., 2022). The gut flora and its byproducts play a major role in the development and incidence of disease (Zhou et al., 2020; Ibrahim et al., 2022). When dietary fiber is broken down and fermented by the beneficial bacteria in the colon, a particular type of metabolite known as SCFAs is produced (Wallimann et al., 2021). It has been found that SCFAs have immunomodulatory attribution, and there is a close relationship between immune function and bone homeostasis. It has been demonstrated that propionate (C3) and butyrate (C4) induce metabolic reprogramming in osteoclasts, which downregulates the expression of osteoclast genes such as TRAF6 and NFATc1 and promotes glycolysis at the expense of oxidative phosphorylation (Lucas et al., 2018). Osteocytes are impacted by numerous immune cells, either directly or indirectly, through OPG/RANKL and other mechanisms. In a normal state, osteoblasts secrete RANKL, a cytokine receptor activator of NF-κB ligand, which precisely controls osteoclast activity (McDonald et al., 2021). Numerous signaling molecules increase the synthesis of RANKL, which binds to RANK on osteoclast precursors to promote osteoclast development; These molecules include TNF-α, IL-6, IL-17, and others (Fischer and Haffner-Luntzer, 2022). By stimulating the synthesis of SCFAs, gut flora may reduce intestinal inflammation and thereby osteoporosis.

According to traditional Chinese medicine theory, Shengu granule are a commonly prescribed medication for treating PMOP of spleen deficiency and dampness trapped type. It also has the effect of strengthening tendons and bones and tonifying the kidney and spleen (Xuyan et al., 2023).

The active components of Shengu granules extract include Luteolin, Sitosterol, Soybean Sterol, Kaempferol, Icariin, and others. Of these, Quercitrin plays a significant regulatory role in bone transformation through a variety of action mechanisms, such as stimulating and inhibiting the osteocyte population, inhibiting matrix metalloproteinase (MMP-9), controlling mesenchymal cell differentiation, and controlling gene expression (RANK; Inchingolo et al., 2022). Mitogen-activated protein kinase (MAPK), NF-ĸb, Wnt/β-catenin, and bone morphogenetic protein2/SMAD (BMP2/SMAD) signaling pathways are all impacted by Luteolin, Kaempferol, and other Flavonoids. Apoptosis pathways also have an impact on bone remodeling (Ramesh et al., 2021).

Zhong Zhuobei’s clinical research has demonstrated that Shengu granules can boost calcium and phosphorus levels, which in turn increases bone metabolism and density (Bei, 2019), however exact mechanism of action of these granules is yet unknown. In our clinic, we found that PMOP patients in South China are often accompanied by gastrointestinal problems, and taking Shengu granules can frequently decrease gastrointestinal problems while improving PMOP, so we hypothesize that Shengu granules may improve PMOP through the gut-bone-immune axis.

## 2 Animals and materials

### 2.1 Production of Shengu granules

Shengu granule herbs were purchased from Guangdong Second Traditional Chinese Medicine Hospital. Prescription as follows:

Dangshen (Dangshen, Lot No.220300721, Origin: Gansu, China),
Gusuibu (Gusuibu, Lot No.220100081, Origin: Zhejiang, China),
Baizhu (Baizhu, Lot No.220160791, Origin: Zhejiang, China),
Xuduan (Xuduan, Lot No.220201, Origin: Sichuan, China),
Fuling (Fuling, Lot No.211204171, Origin: Hunan, China),
Yiyiren (Yiyiren, Lot No.220403291, Origin: Guizhou, China),
Huangbai (Huangbai, Lot No.220103871, Origin: Sichuan, China),
Cangzhu (Cangzhu, Lot No.2203005, Origin: Neimenggu, China),
Niuxi (Niuxi, Lot No.221202641, Origin: Sichuan, China),
Mudanpi (Mudanpi, Lot No.211200139, Origin: Anwei, China),
Shanzhuyu (Shanzhuyu, Lot No.211001601, Origin: Zhejiang, China),
Yanhusuo (Yanhusuo, Lot No.220107, Origin: Zhejiang, China),
Biejia (Biejia, Lot No.220305732, Origin: Hubei, China).

The above herbs by ultra-centrifugation, ultrafiltration to remove impurities, made Shengu granule concentrated granules.

### 2.2 Animal models and group

12-week-old Sprague–Dawley female rats (SCXK(YUE)2013-0002) were purchased from the Guangdong Medical Laboratory Animal Center (Lot No.44007200100399), which were housed at a specialized pathogen-free environment with conventional laboratory settings (25°C ± 2°C, 50% ± 5% humidity, and a 12/12 h light/dark cycle). Following a week of adaptation to their new surroundings, Three groups (*n* = 12 per group) were randomly assigned to all the mice: the Sham group, the Ovariectomy (OVX) group, and the SG group. Sham group is as blank control group, OVX group is as PMOP model group, and SG group is as taking Shengu granules group. Rats in the SG and OVX groups underwent bilateral ovariectomy, while the rats in the Sham group only underwent removal the same size of fat near the ovary. Beginning on the tenth day after the bilateral ovariectomy procedure and ending with sacrifice, the rats in the SG group were given Shengu granules (12 g/kg.d) orally for 14 weeks. Rats were administered the concentration using the conversion method of body surface area, which was based on the concentration of Shengu granules used in the clinic for adults weighing 60 kg. Rats in both the Sham group and the OVX group were given the same volume of regular distilled water. Rats’ body weight was recorded once a week, and the body surface area was used to modify the gavage dosage. Every group of rats had unrestricted access to food and water. Following a 14-week treatment period, the rats underwent a 12-h fast, followed by weighing and an injection of a 2% sodium pentobarbital solution (40 mg/kg). All rats’ femur, colon tissue, serum, and feces samples were collected for further analysis.

### 2.3 Chemicals and reagents

The purity of the standards: Acetic acid (Aladdin, United States), Butyric acid (Sigma, United States), Hexanoic acid (Dr. Ehrenstorfer, United States), Isobutyric acid (Toronto Research Chemicals, Canada), Isovaleric acid (Shanghai yuanhe Bio-Technology Co., Ltd., China), Pentanoic acid (NU-CHEK, United States), Propionic acid (Sigma, United States), is above 99% according to the gas chromatography (GC) assay.

Other chemical reagents: Methyl alcohol (Fisher, United States), Purified water (Hangzhou Wahaha Group Co., Ltd., China), Formic acid (Fisher, United States), Acetonitrile (Fisher, United States), 3-nitrophenylhydrazine hydrochloride (Sigma, United States), N-(3-dimethylaminopropyl)-N′-ethylcarbodiimide hydrochloride (Aladdin) are at chromatographic grade purity.

### 2.4 Biochemical analysis

Commercial ELISA kits were used to measure the serum levels of bone turnover markers (BTMs) and inflammatory markers. The kits used in this study were ELISA for blood β-CTX and PINP (Elabscience Biotechnology Co., Ltd., China). Additionally, the serum inflammatory markers like TNF-α, TGF-β, IL-10 (Elabscience Biotechnology Co., Ltd., China), and IL-17 (ELK Biotechnology Ltd., China) were included.

### 2.5 Bone analysis

The mouse right femurs were collected and removed the muscles around the bone. After fixed with 4% paraformaldehyde solution, the right femurs were fixed in a Micro-CT sample detection plate. A Quantum GX μ-CT system (Bruker micro-CT, United States) scanned them. To obtain sequential planar μCT images of the distal femur, the distal femur was scanned at a resolution of 10 μm along its long axis at an angle of 360°. After scanning, a 1.5 mm area of the distal femur was selected as the region of interest on the mainframe. Three-dimensional image reconstruction of the trabecular were performed, which were analyzed by the DataViewer software.

### 2.6 Analysis of gut microbiota sequencing

Fresh fecal samples per group were gathered and put in liquid nitrogen-sealed tubes. According to the manufacturer’s instructions, the HiPure fecal DNA kit (Ma River, Guangzhou, China) was used to extract DNA from feces samples, and gel electrophoresis was used to identify the bacterial genomic DNA. The study of 16S rDNA genes was used to identify the composition of the gut flora. Through the use of barcode-specific primers during sample DNA amplification, the 16S V3-V4 rRNA region of bacteria was enriched. Test every sample twice in parallel. The UPGMA technique is used to cluster the data and create a sample clustering tree so that the similarity between various samples can be examined. The PICRUSt 2 program projected the community’s function, and the KEGG pathway data of the genes was used to estimate the pathway state of the entire community. Detailed instructions for sequencing rat feces are available in Supplementary material.

### 2.7 SCFAs analysis

The SCFAs (Acetic acid, Butyric acid, Hexanoic acid, Isobutyric acid, Isovaleric acid, Pentanoic acid) in the feces of the rats were identified by GC analysis. Gas chromatography is performed after combining the proper amounts of feces by centrifuging and spinning five times the volume of water. After that, the supernatant was combined with one-tenth of the formic acid’s volume. A 0.45 μm-diameter microporous membrane was used to filter the mixture. The Nexera High-performance liquid chromatography LC-30A gas chromatograph was used to fill the filter. A fused silica gel column (100*2.1 mm, 1.7 μm, ACQUITY UPLC BEH C18, Waters Corporation) was utilized to separate SCFAs. To obtain a standard curve for SCFAs, we carried out a comprehensive methodological analysis. The target’s peak size, peak form, and retention time were all measured in addition to its peak area. The Supplemental material include the detailed procedures.

### 2.8 Histological evaluation

After the colon was fixed in 10% neutral formalin fixative for 48 h, the tissues were routinely dehydrated, embedded in paraffin, sectioned, and stained with H&E. the H&E-stained sections of the colon tissues were scored for histological damage. The evaluation was done in the dark. Three criteria were used to determine the score: the severity of inflammation (0–3: none, mild, moderate, severe), degree of damage (0–3: none, mucosal, mucosal and submucosal, transmural), and crypt damage (0–4: none, 1/3 basal invasion damage, 2/3 basal invasion damage, surface epithelium intact only, loss of the entire crypt and epithelium). The scores were finally summed up as the histological damage score for H&E stained sections. Supplementary Table 1 contain a detailed explanation of the scoring rules.

### 2.9 Immunofluorescence

Colon sections were paraffin dewatered and restored for antigen. Sections were rinsed with PBS, incubated overnight at 4°C with FOXP3 antigen (1:200; Servicebio Technology Ltd., China), and then incubated with secondary antibody (Servicebio Technology Ltd., China) for 30 min at 37°C. Sections were rinsed again with PBS and color developed by adding a drop of DAB colorant. Sections were washed again with PBS and color developed dropwise with DAB color solution. Colon sections were counterstained with hematoxylin at 25°C for 30 s. The expression of FOXP3 in the colon was observed. A fluorescence microscope (Olympus BX43 + DP27, Japan) was used for observation under different wavelengths of light sources.

### 2.10 Western blotting

The total protein of colon and femur tissue were separately extracted using Rapid In-Process Lysis of Cellular Tissues (RIPA). The BCA technique, Protein Assay Kit (Thermo Technology Ltd., United States), were used to determine the protein concentration. Gel preparation was performed according to the Accutane Gel Rapid Preparation Kit. Protein samples were divided, a polyvinylidene difluoride membrane was transferred, and the membrane was sealed. Total protein samples extracted from colon tissue were incubated with ZO-1 (Servicebio Technology Ltd., China) and Occludin (Servicebio Technology Ltd., China) primary and secondary antibodies, and total protein samples extracted from femur tissue were incubated with OPG (Servicebio Technology Ltd., China) and RANKL (Servicebio Technology Ltd., China) primary and secondary antibodies. Then they were exposed to color development, and the intensity of the bands was quantified by ImageJ software, which was used for data analysis.

### 2.11 Statistical analysis

The data was reported as mean ± SD. The statistical software SPSS 25.0 was used for the analyses. One-way ANOVA was used to analyze statistical differences, and the Tukey test was then performed. Pearson’s correlation was used to show the correlations among the parameters. *p* < 0.05 was deemed statistically significant.

## 3 Result

### 3.1 The effect of Shengu granules on the expression of bone microarchitecture, bone metabolism biomarkers, and inflammatory factors

**Figure 1:**
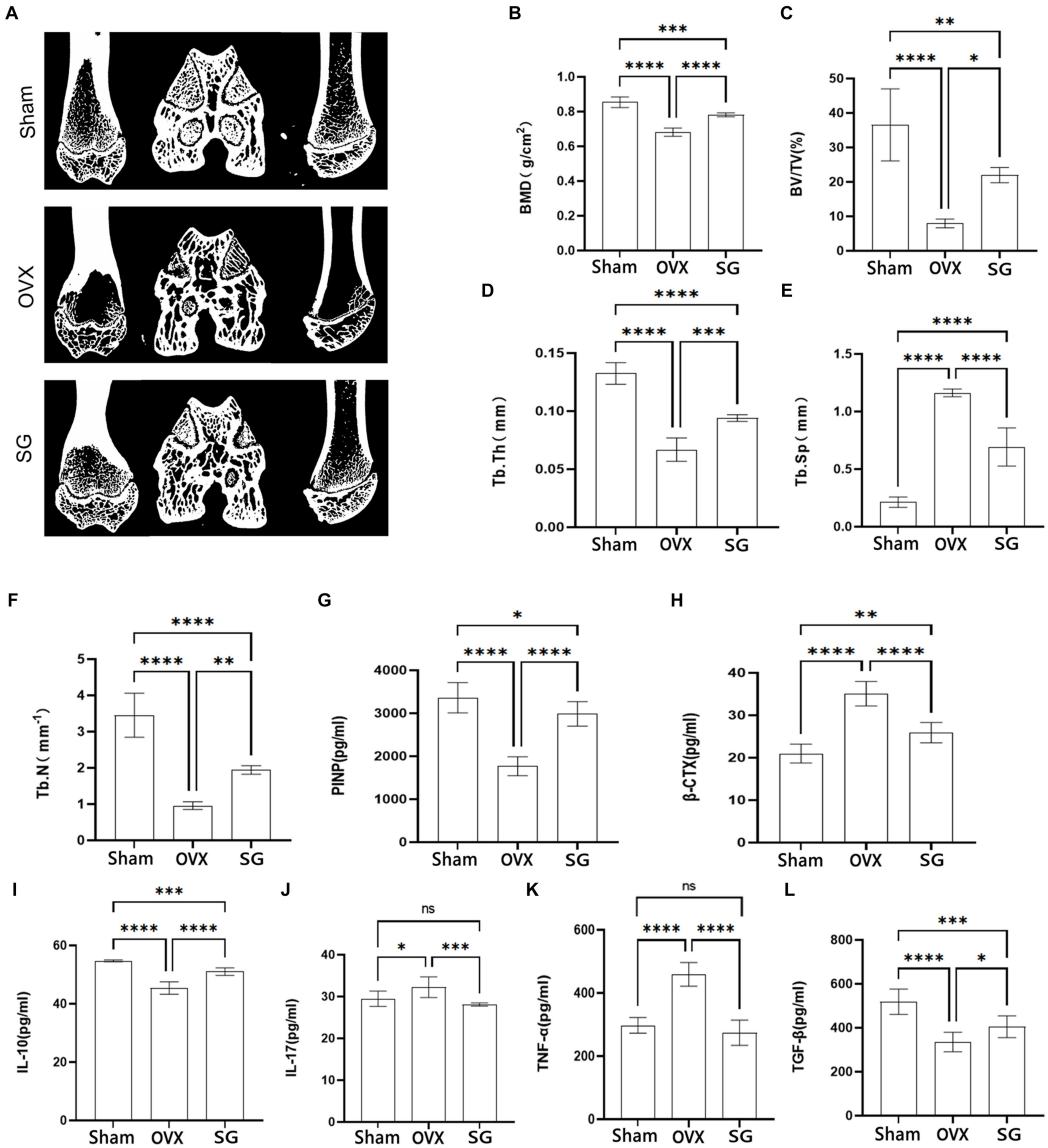
The effect of Shengu granules on the expression of bone microarchitecture, bone metabolism biomarkers and inflammatory factors. Transverse, sagittal, and coronal views of every group rats femur micro-CT **(A)**. Bone mineral density **(B)**, bone volume/tissue volume ratio **(C)**, average thickness of bone trabecular **(D)**, average width of medullary cavity between trabecular bone **(E)**, number of trabecular bone **(F)**. Content of PINP **(G)**, β-CTX **(H)**, IL-10 **(I)**, IL-17 **(J)**, TNF-α **(K)**, and TGF-β **(L)**, in serum. *n* = 7, ^*^p < 0.05; ^**^p < 0.01; ^***^p < 0.001; ^****^p < 0.0001. Data are represented as mean ± SD.

The micro-CT results of femur (Figure 1A) showed that the bone trabeculae in Sham group were homogeneously spread, arranged regularly, organized and intertwined into a mesh. The OVX group’s bone trabeculae had an elongated shape, were loosely organized, and had a notable decrease in both number and density as well as an increase in luminal space. After the intervention of Shengu granules, the number and shape of the bone trabeculae were remarkably increased compared with OVX group (Figures 1B–F). The SG group enhanced bone density (BMD) and tissue density (TMD) compared to the VOX group (Figure 1B). Comparing SG group to OVX group, SG group’s the ratio of bone volume/tissue volume (BV/TV) increased (Figure 1C), and the proportion of bone trabecular area enhanced (Figure 1F). The concentrations of pro-inflammatory factors, IL-17 and TNF-α, significantly decreased in the SG group compared to the OVX group (Figures 1J,K, Sham), and the concentrations of anti-inflammatory factors IL-10 and TGF-β, increased remarkably in SG group relative to OVX group (Figures 1I,L), which showed that Shengu granules could regulate the balance of pro-inflammatory and anti-inflammatory factors. Additionally the bone formation marker PINP, increased significantly in SG group compared to OVX group (Figure 1G), and the bone degeneration marker, β-CTX, was notably decreased in SG group compared to OVX group (Figure 1H). In summary, Shengu granules can increase the density of bone trabeculae and decrease osteoporosis, and at the same time, the anti-inflammatory factors are raised and the pro-inflammatory factors are decreased.

### 3.2 The effect of Shengu granules on OVX rats’ intestinal microbiota

**Figure 2:**
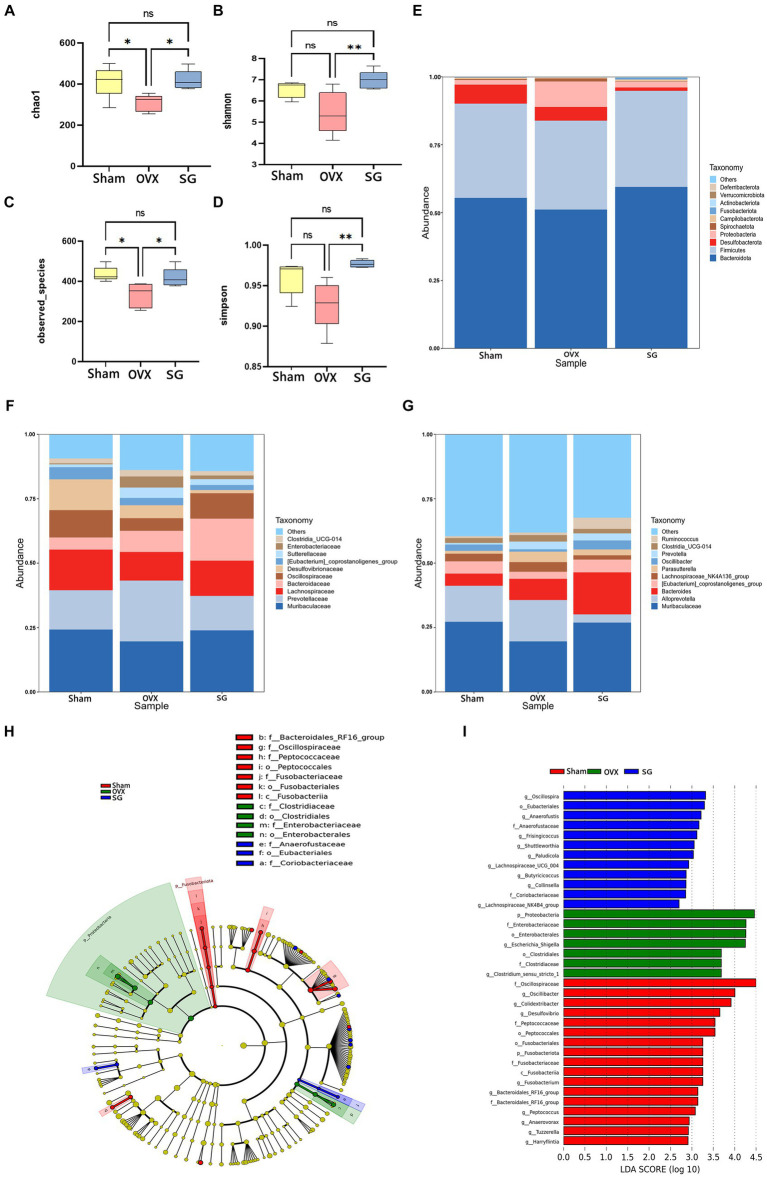
The effect of Shengu granules on OVX rats’ intestinal microbiota. The diversity indices, such as Chao1 **(A)**, Shannon **(B)**, observed_species **(C)** and Simpson **(D)**; 15 most abundant bacteria at phylum **(E)**, family **(F)** and genus **(G)** levels. Cladogram **(H)**: node diameter size is in proportion to relative abundance size, node in each layer represents phylum/class/order/family/genus from inside to outside. LDA **(I)** score diagram: the Sham group is red bars indicate species with relatively high abundance, the OVX group is green bars indicate species with relatively high abundance, and the SG group is blue bars indicate species with relatively high abundance. *n* = 5, ^*^p < 0.05; ^**^p < 0.01. Data are represented as mean ± SD.

The alpha diversity is calculated as the diversity index of the sample flora, which is commonly expressed as Chao1, Shannon, Simpson and observed_species. Chao1 represents the total number of species (Figure 2A). The total number of flora in the OVX group was significantly lower than that in the Sham group, while the Shengu granules group was significantly higher than the OVX group (Figure 2A). observed_species indicates species type (Figure 2C). The OVX group had significantly lower flora species than the Sham group, while the SG group was significantly higher than the OVX group (Figure 2C). Simpson and Shannon indicated the diversity of microbial species (Figures 2B,D). The diversity of flora in the OVX group was significantly lower than that in the Sham group, while the SG group was significantly higher than the OVX group (Figures 2B,D). Therefore Shengu granules can recover the structure of gut microbiota to normalization.

In order to explore the ways in which Shengu granules influence certain modifications in the gut microbiota, we analyzed the top 15 bacterium at various classification levels. At the phylum level, as shown in Figure 2E, the trend of Bacteroidota, Firmicutes, Proteobacteria and Desulfobacterota in the SG group was the same as that in the Sham group. Of these, Bacteroidota, Firmicutes and Proteobacteria increased in comparison to the OVX group, while Desulfobacterota decreased. Bacteroidota, Firmicutes were the most abundant phylum among all microbial groups in fecal samples. It is well known that the ratio of Firmicutes to Bacteroidota (F/B ratio) is used as a commonly used parameter to assess the influence of gut microbes in many diseases (such as obesity, gene expression). It was found that the OVX group had a remarkably higher F/B ratio compared to the SG group. At the family level, as shown in Figure 2F, the change trend of Muribaculaceae, Prevotellaceae, Lachnospiraceae, and Oscillospiraceae in SG group is similar to that in Sham group. In OVX group, the relative abundances of Muribaculaceae, Lachnospiraceae, and Oscillospiraceae were lower than those of the Sham and SG groups, and the relative abundances of Bacteroidaceae in SG group were higher than those in Sham and OVX groups. At the genus level, as shown in Figure 2G, Muribaculaceae, [Eubacterium]_coprostanoligenes_group decreased in OVX group compared to Sham group, while SG group reversed these changes, the relative abundance of Alloprevotella was much lower in SG group than in Sham and OVX groups.

To further analyze the significant bacterial community markers of postmenopausal osteoporosis regulated by Shengu granules, LefSe analysis (Linear discriminant analysis Effect Size) was performed on the results (Figures 2H,I), and there were 16 dominant bacterial communities in the Sham group: f_Oscillospiraceae, g_Oscillibacter, g_Colidextribacter, g_Desulfovibrio f_Peptococcaceae, o_Peptococcales, o_Fusobacteriales, p_Fusobacteriota, f_Fusobacteriaceae, c_Fusobacteriia, g_Fusobacterium, f_Bacteroidales_RF16_group, g_Peptococcus, g_Anaerovorax, g_Tuzzerella, g_Harryflintia. There were 7 dominant bacterial groups in the OVX group, including p_Proteobacteria, F_Enterobacteriaceae, o_Enterobacterales, g_Escherichia_Shigella and o_Clostridiales f_ Clostridiaceae, g_Clostridium_sensu_stricto_1. The SG group has 11 dominant bacterial groups: g_Oscillospira, o_Eubacteriales, g_Anaerofustis, f_Anaerofustaceae, g_Frisingicoccus g_Shuttleworthia,g_Lachnospiraceae_UCG_004,g_Butyricicoccus,g_Collinsella, f_Coriobacteriaceae, g_Lachnospiraceae_NK4B4_group. And 11 genera in the SG group can be used as biomarkers for Shengu granules to improve osteoporosis.

### 3.3 The effect of Shengu granules on the production of SCFAs

**Figure 3:**
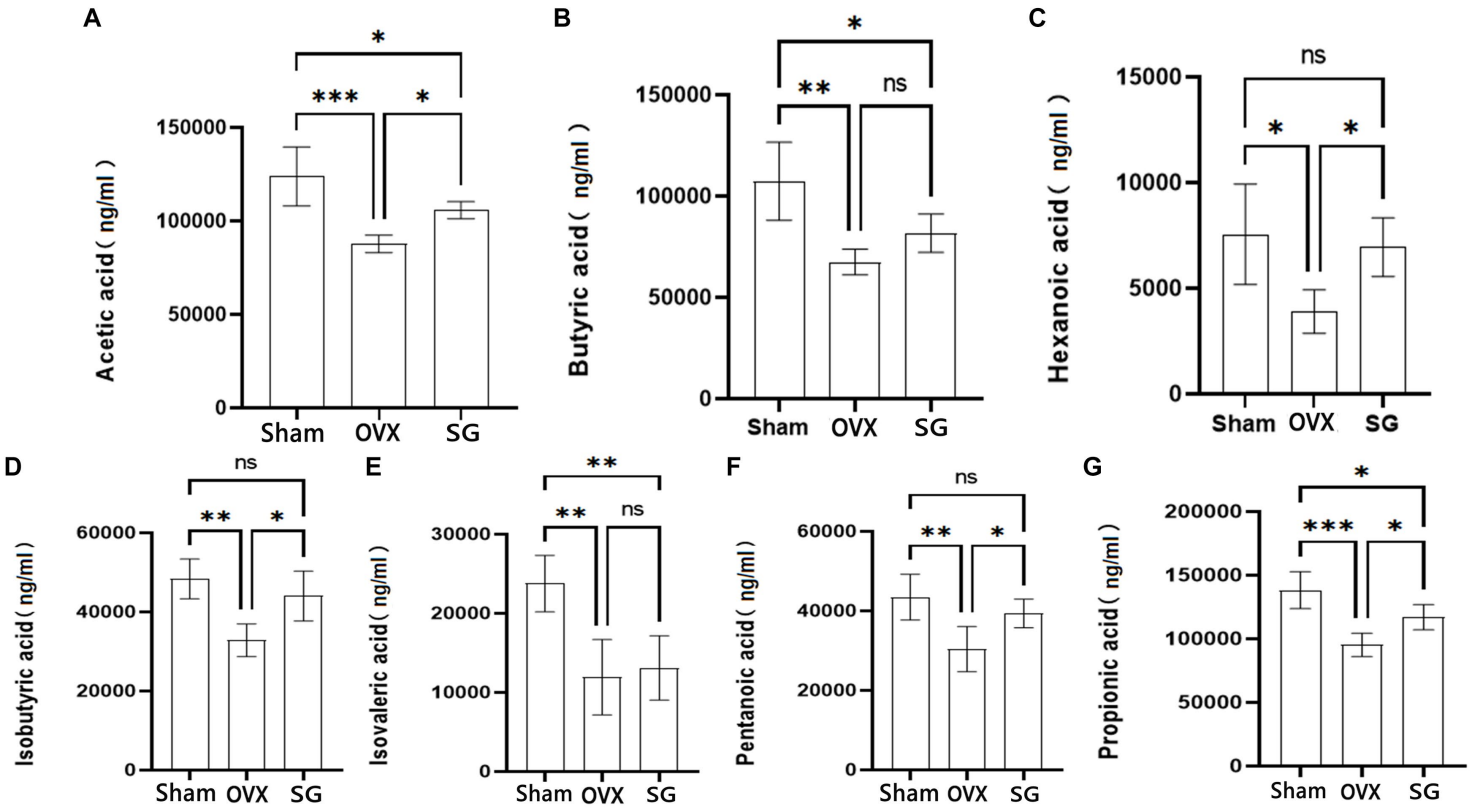
The effect of Shengu granules on the production of SCFAs. Acetic acid **(A)**, Butyric acid **(B)**, Hexanoic acid **(C)**, Isobutyric acid **(D)**, Isovalerate acid **(E)** and Pentanoic acid **(F)**, Propionic acid **(G)** in the feces of rats. *n* = 5, ^*^p < 0.05; ^**^p < 0.01; ^***^p < 0.001. Data are represented as mean ± SD.

SCFAs are important metabolites of intestinal microorganisms, which not only help intestinal mucosal cells to convert energy and promote cellular metabolism (van der Hee and Wells, 2021), but also regulate various inflammatory responses in intestinal epithelial cells and intestinal mucosal tissues, thus maintaining intestinal barrier homeostasis. In our study, we found that the OVX group decreased the content of SCFAs compared with the Sham group. Besides, the SG group increased the content of SCFAs compared with the OVX group, Which showed different degrees in Acetic acid, Butyric acid, Hexanoic acid, Isobutyric acid, Isovaleric acid, Pentanoic acid and Propionic acid (Figure 3). The administration of Shengu granules improved such decreases to different degrees. This indicates that the administration of Shengu granules can reverse the changes in SCFAs.

### 3.4 The effects of Shengu granules on intestinal barrier, FOXP3 cells and OPG/RANKL signaling pathway protein expression

**Figure 4:**
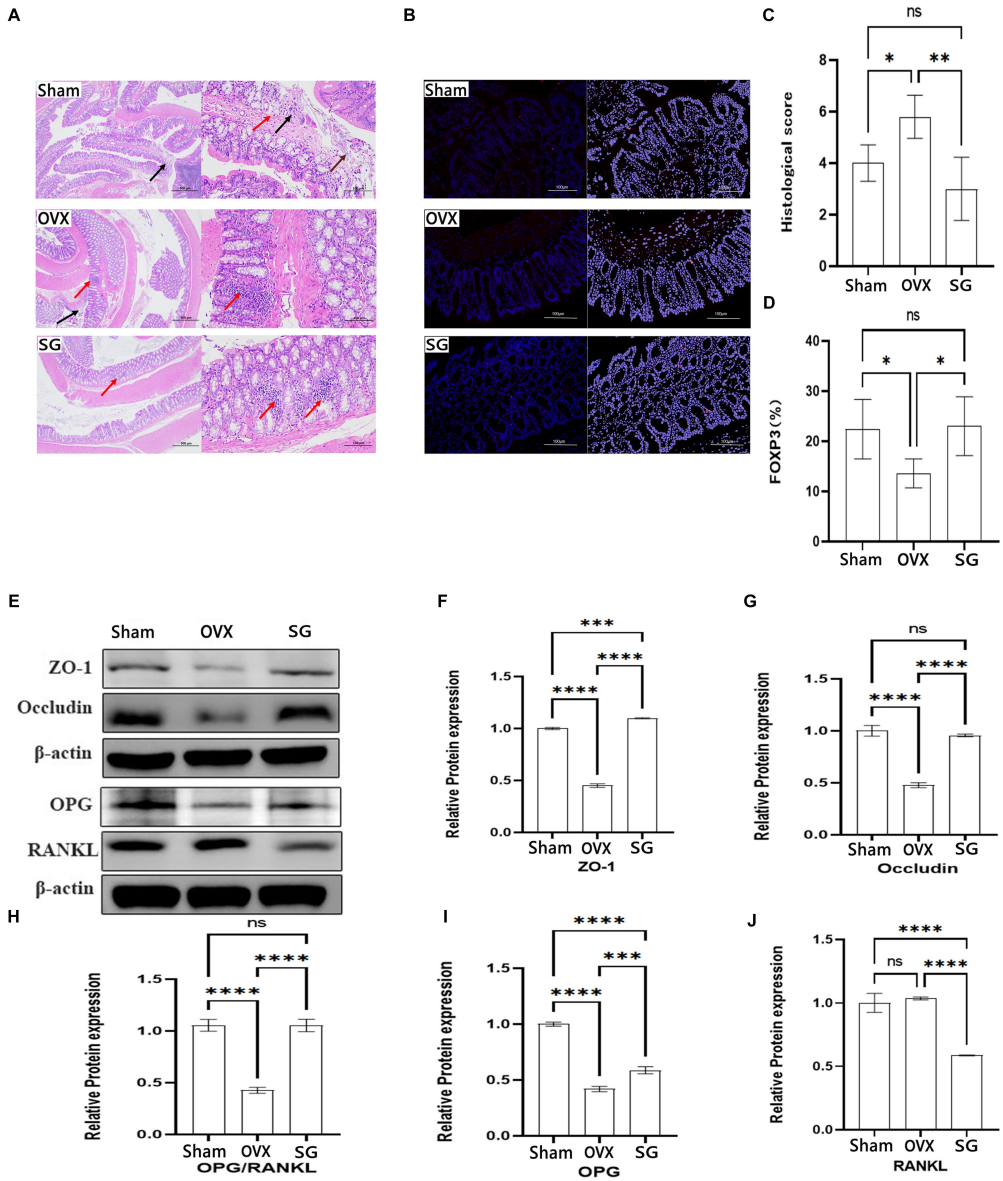
The effect of Shengu granules on intestinal barrier, FOXP3 cells and OPG/RANKL Signaling pathway protein expression. The colon pathological sections of the Sham, OVX and SG group, the former one (magnification, 40X), the latter one (magnification, 200X) **(A)**. Necrosis and exfoliation of mucosal epithelial cells (brown arrow), lymphocyte infiltration (red arrow), and nuclear fragmentation (black arrow) in Sham group; Nuclei were fragmented (black arrow), and more lymphocytes were found in the lamina propria (red arrow) in OVX group. Small focal infiltration of lymphocytes (red arrows) in SG group **(A)**. The Immunofluorescence results of each group (magnification, 200X) **(B)**. Histological score **(C)**. Percentage of mean Immunofluorescence intensity of FOXP3 cells in the three groups **(D)**. Protein blot analysis, semiquantitative analysis of protein blotting results of ZO-1 and Occludin in colon biopsy, OPG and RANKL in tibia tissue **(E)**. The ZO-1 and Occludin protein expression in colon tissue **(F,G)**. The OPG and RANKL protein expression ratio **(H)**. The OPG and RANKL protein expression in tibia tissue **(I,J)**. Statistical significance was evaluated using one-way ANOVA procedure and Tukey test. Different letters represent significant differences between groups (*p* < 0.05) vs. OVX group. *n* = 5, ^*^p < 0.05; ^**^p < 0.01; ^***^p < 0.001; ^****^p < 0.0001. Data are represented as mean ± SD.

In recent years, relevant studies have found (Zhou et al., 2023) that the decline in human bone mass is frequently associated with the deterioration of the intestinal barrier and the increasing in inflammatory substances generated by the immune system. In our study, representative H&E-stained histological sections and histopathology showed focal erosions and a higher amount of lymphocyte infiltration in HE-stained intestinal tissue sections in OVX group compared to SG group, accompanied by significant epithelial cell damage/loss or cellular mucin consumption (Figure 4A). According to the colitis score, Shengu granules repaired ovariectomy-induced morphological damage to colonic tissue (*p* < 0.05; Figure 4C). FOXP3 regulatory T (Treg) cells maintain immune homeostasis by producing anti-inflammatory factors like IL-10 and the transforming growth factor TGF-β to suppress excessive immune responses, and their dysregulation has been associated with a wide range of human diseases, such as autoimmune disorders allergies and cancer (Jung et al., 2017). FOXP3 cells expression were notably lower in the OVX group compared to the Sham group (Figures 4B,D). FOXP3 cells were remarkably higher in the SG group compared to the OVX group (*p* < 0.05; Figure 4D). From this we learn about that Shengu granules can promote the repair of FOXP3 cells in the colon epithelium.

According to previous studies, postmenopausal osteoporosis patients develop an imbalance of intestinal flora, which often manifests the destruction in the intestinal structure and a risen trend in inflammatory indicators. We chose to observe the effect of Shengu granules on intestinal structure by using structural connectivity proteins ZO-1 and Occludin in colon biopsy. From the data, we can know about that ZO-1 and Occludin protein expression of the Sham group was remarkably higher than the OVX group (Figures 4F,G). Besides, ZO-1 and Occludin protein expression of the SG group was remarkably higher than the OVX group, which shows that Shengu granules can restore intestinal barriers by reversing intestinal structural disruption. OPG/RANKL is an important pathway for the induced osteoblastic cell differentiation. And after restoring the intestinal barrier by taking Shengu granules, the OPG protein expression of SG group was elevated compared with OVX group, and the RANKL protein expression of SG group was remarkably reduced compared to OVX group (Figures 4I,J). OPG could competitively bind with RANK, blocking the interaction between RANKL and RANK, thus inhibiting the activation, differentiation and maturation of osteoclasts (Kenkre and Bassett, 2018). As we all know, the ratio of OPG and RANKL protein expression plays an important role in osteoclast differentiation. The ratio of OPG and RANKL protein expression of SG group was remarkably higher compared with OVX group (Figure 4H). Apparently, Shengu granules increased the OPG and decreased the RANKL protein expression. Therefore, Shengu granules reversed the intestinal barrier disruption caused by ovariectomy, reduced intestinal inflammation, and decreased bone protein loss (Figure 4E).

### 3.5 Correlation analysis between important differential intestinal species and SCFAs, immune factors, and bone metabolism

**Figure 5:**
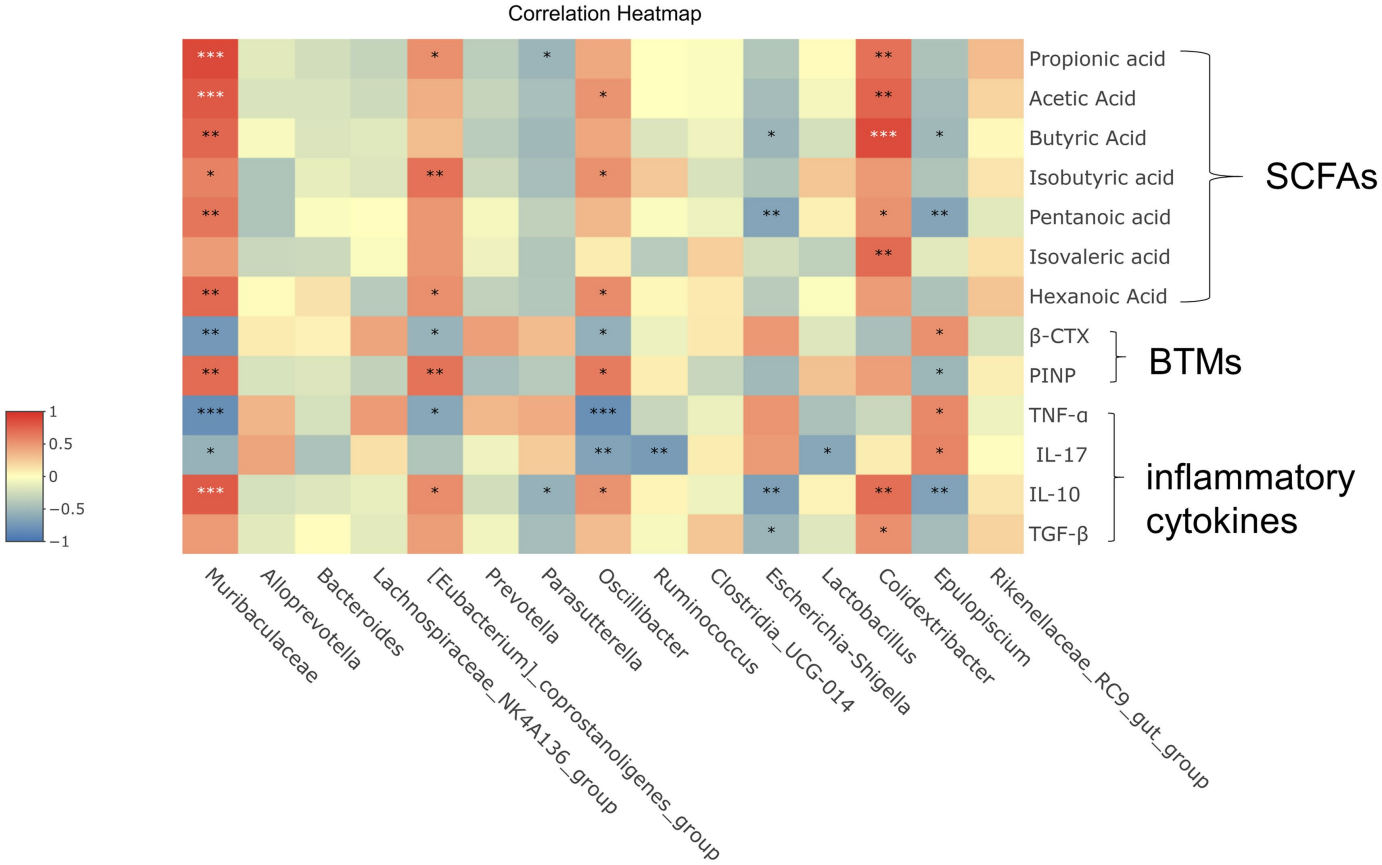
Correlation analysis between important differential intestinal species and SCFAs, immune factors, and bone metabolism. Heat map shows the correlation values between important differential intestinal species and biomarkers. Correlation coefficient |R| ≥ 0.5 indicates strong correlation. Red indicates a positive correlation and blue indicates a negative correlation. The darker the color, the stronger the correlation. Each row shows information about the bacterial taxa (phylum, class, family, and genus).

Overall, these results indicated that Shengu granules regulated the gut microbiota in OVX rats (Figure 5). To further understand the potential role of each essential gut microbiota, pearson correlation analysis was performed to verify the essential flora, SCFAs, inflammatory factors and bone metabolism.

In these species, [Eubacterium]_coprostanoligenes_group and Isovaleric acid, PINP had strong positive correlation (|R| ≥ 0.5 or higher). Muribaculaceae was significantly positive correlated with Propionic acid, Acetic acid, Butyric acid, Isovaleric acid, Pentanoic acid, Hexanoic acid, PINP and IL-10, while shown a quite strong negative correlation (|R| ≥ 0.5 or higher) with β-CTX, TNF-ɑ and IL-17. It is shown that there was a noticeable positive correlation (|R| ≥ 0.5 or higher) between Clostridiacea and Propionic acid, Acetic acid, Butyric acid, Isovaleric acid, Pentanoic acid, IL-10. Between Oscillibacter and TNF-ɑ, IL-17 shown a quite strong negative correlation (|R| ≥ 0.5 or higher). When considered collectively, these results suggest that correlation analysis further reveals that potential significant bacterial changes play a significant role in improving osteoporosis which induced by Shengu granules, and that further studies are necessary to figure out how these bacterium play the effect on the host and participate in amelioration osteoporosis.

## 4 Discussion

Up to now, Shengu granules have been used and about 7,000 people take them every year in the Guangdong Provincial Second Hospital of Traditional Chinese Medicine, China. In the preliminary clinical trial, we collected clinical data to provide objective data support for verifying the role of Shengu granule in improving PMOP symptoms and bone metabolism, but the mechanism has not yet been clarified. The study of the intestinal flora and its metabolites in osteoporotic rats received Shengu granules, we confirmed that Shengu granules associated with regulation of gut microbiota and its metabolite SCFAs to restore intestinal immune-skeletal signaling axis, thus improving bone loss.

The homeostasis of PMOP is closely related to the intestinal flora and also has a complex relationship with inflammatory cytokines. More and more evidence proves the gut-bone-immune axis is an important research direction in osteoporosis (Chen et al., 2023). As we all know, the treatment of PMOP is mainly based on the administration of western medicines such as calcium agents that promote bone growth. The drugs that promote osteogenesis and inhibit bone resorption in order to control the occurrence of osteoporosis. With the in-depth study of Chinese traditional medicine, there are many studies show that Chinese herbal formulations can restore the intestinal barrier and thus ameliorate osteoporosis by the gut-bone-immune axis (Tang et al., 2021; Sun et al., 2022; Xie et al., 2022). This study focused on the anti-osteoporosis mechanism of Shengu granule by the gut-bone-immune axis.

In our results, we analyzed that the structural populations of intestinal flora of rats taking Shengu granules differed from the rats in OVX group, and the diversity of intestinal microbial communities were increased after taking Shengu granules, and at the same time osteoporosis indexes in the SG group were decreased compared with the OVX group. According to the LefSe results, we analyzed the changes of significant bacterium in each group. Proteobacteria, Enterobacteriaceae, Enterobacterales, Escherichia_Shigella are intestinal pathogenic bacterium, these pathogenic bacteria can trigger inflammatory response, which will lead to intestinal disruption of the mucosal barrier in OVX group (Lupp et al., 2007; Veziant et al., 2016; Peng et al., 2020). This suggests that Shengu granules can reduce intestinal inflammatory bacteria in osteoporotic rats. Eubacteriales, the dominant group in SG group, is a core group of Gram-positive bacteria in the intestinal tract, which Bacterium affiliated may produce large amounts of butyrate, propionate, and acetate. In addition, Oscillibacter is strongly associated with obesity and intestinal diseases, which is the main microorganism producing butyrate, and inversely associated with the development of colitis. An increase in the relative abundance of Oscillibacter positively ameliorates the inflammatory response and attenuates osteoporosis (Ng et al., 2014; Gamallat et al., 2019; Xu et al., 2019, 2021). Oscillospira, a plentiful component of the human gut flora, can decrease inflammation response through fermentating the complex of plant carbohydrates to produce butyrate, thereby ameliorating the inflammatory response (Carding et al., 2015). Lachnospiraceae family are abundant and widespread bacteria in the intestinal microbiota of healthy individuals, and they are the main butyrate producers (Hamer et al., 2008). In turn, butyrate is an essential energy source for intestinal epithelial cells, which strengthens the intestinal epithelium and reduces the risk of inflammation and carcinogenesis (Zhang et al., 2021). In a related study, Lachnospiraceae_UCG_004 was found to increase the concentration of intestinal SCFAs (Ma et al., 2022). Butyricoccus is a probiotic known bacteria to produce SCFAs, especially butyrate, and has significant intestinal immunomodulatory property, which can significantly ameliorate intestinal damage and intestinal mucosal disorders; Thus Butyricoccus has become a popular bacterial group to analyze the host’s intestinal stationary for beneficial effects (Houtman et al., 2022). Eubacteriales, Oscillibacter, Oscillospira, Lachnospiraceae_UCG_004 and Butyricoccus are as the important component of the intestinal flora in the SG group. They are the dominant flora that promote the production of SCFAs. Apparently, the Shengu granules may promote an increase in the number of dominant flora that produce SCFAs. SCFAs interact with G-protein-coupled receptors to regulate inflammation, intestinal barrier integrity, glucose response, energy homeostasis, and other host responses (van der Hee and Wells, 2021). Additionally, SCFAs can be utilized to lower the risk of intestinal inflammation by inhibiting NSAIDs, which have the potential to trigger inflammatory reactions in the gastrointestinal tract; Specifically, SCFAs reduce inflammation mainly by blocking the NF-κB pathway and/or histone deacetylase function (HDACI; Lucas et al., 2018). This lead to the downregulation of pro-inflammatory molecules like TNF-α, IL-6, IL-12, and IFN-γ and the upregulation of anti-inflammatory factors like IL-10 and TGF-β (Mukherjee et al., 2020). Therefore, the ability of Shengu granules to promote the production of SCFAs by intestinal flora may be a potential mechanism for Shengu granules to ameliorate inflammation response.

Furthermore, SCFAs produced in the gut which have immunomodulatory and anti-inflammatory capabilities, inhibit pathogen growth by maintaining low pH, and stimulate butyrate-producing bacteria to enhance the intestinal immune barrier (Smith et al., 2013). Butyrate regulates IL-10 receptor junction protein claudin-1 transcription, decreases the permeability of epithelial tissue, and increases the thickness of the intestinal mucosal layer (Wang et al., 2012; Zheng et al., 2017). Considering the results of intestinal sections, epithelial cell damage/loss or cellular mucin consumption in the OVX group provided evidence for inflammatory factor production and intestinal barrier disruption. The SG group greatly decreased the damage to the intestinal mucosal barrier compared to the OVX group, because the increasing of SCFAs in the intestines and protein expression of Occludin (membrane integrative protein) and ZO-1 (scaffolding protein). In addition, pro-inflammatory factors like IL-17 and TNF-α were reduced, and anti-inflammatory factors like TGF-β and IL-10 were elevated, which decrease inflammatory responses. Shengu granules play a significant role in reducing inflammatory response and restoring intestinal barrier. In the gut-bone-immune axis, FOXP3 is an important cytokine that promotes the transformation of Treg, which is an anti-inflammatory and pro-inflammatory factor (Round and Mazmanian, 2010). Some studies have found that the production and function of Treg are closely related to the regulation of bacterial flora, whereas SCFAs promote the differentiation of naïve T-lymphocytes into Treg (Round and Mazmanian, 2010; Furusawa et al., 2014; Park et al., 2015). Some related studies have shown that butyrate and propionate increase the acetylation of the FOXP3 promoter in the transcript; Promoter acetylation is critical for promoting Treg differentiation (Round and Mazmanian, 2010; Furusawa et al., 2014). In the SG group, the rise of the SCFAs content was accompanied by an increase in mucosal regulatory FOXP3 T cells and was consistent with the corresponding upregulation of IL-10. SCFAs decrease IL-17 production in T cells (including Th10, Th1, and Treg cells), and the cytokine milieu directly promote the conversion of naïve T cells into Th1 or Th17 (Park et al., 2015), and IL-17 is a significant cytokine that induces Th17 production (Gaffen, 2011). Bone immune disorders caused by the cellular imbalance of the Th17/Treg axis and the associated cytokines are a major risk factor of osteoporosis (Okamoto, 2017). These may indicate that the Shengu granules may have increased FOXP3 expression regulating treg conversion and increased SCFA regulating Th17 conversion, thus ameliorating the effect of the Th17/treg axis on osteoporosis. According to related studies, the balance of inflammatory cytokines produced by the Th17/Treg axis regulates the OPG/RANKL signaling axis (Yang et al., 2015). The OPG/RANKL ratio is an important differentiation indicator of osteoblast and osteoclast (Boyce and Xing, 2008). The RANKL is blocked and bound by OPG which can enhances osteoblast differentiation and inhibits osteoclast differentiation (Boyce and Xing, 2008). In the SG group, the levels of pro-inflammatory factors IL-17 and TNF-α decreased, the levels of anti-inflammatory factors IL-10 and TGF-β increased, the expression of OPG proteins increased, the expression of RANKL proteins decreased, and the OPG/RANKL ratio increased. The results showed that Shengu granules decreased bone loss by enriching SCFAs-producing probiotics, decreasing intestinal epithelial permeability, and restoring the balance of IL-10 and TGF-β with IL-17 and TNF-α inflammatory factors that regulated the balance of the OPG and RANKL signaling axis. In summary, Shengu granules ameliorate ovariectomy-induced osteoporosis by the gut-bone-immune axis.

Correlation analysis revealed that osteoporosis serum indicators, essential intestinal flora (Muribaculaceae, [Eubacterium]_coprostanoligenes_group, Oscillibacter, Epulopiscium), inflammatory factors, and SCFAs were associated with bone metabolism in the function of Shengu granules. There was a significant relationship among inflammatory factors, SCFAs and bone metabolism. To a certain extent, the anti-osteoporosis mechanism of Shengu granules in the gut-bone-immune axis was verified.

In total, Experimental basis for the clinical application of Shengu granules is provided by the present study in PMOP and demonstrates that modulation of the gut-bone-immune axis may be a potential strategy for the treatment of PMOP. However this study has some limitations. This experiment did not directly measure Treg and Th17 cells, and only verified the relevant inflammatory factors, which may be slightly insufficient experimental data, so we will investigate the effect of Treg/Th17 axis in a follow-up study to add a more strong proof of the experiment data.

## 5 Conclusion

In conclusion, our study found that Shengu granules improved osteoporosis by improving intestinal flora and its metabolites which attenuated intestinal immune response. The experimental results verified that Shengu granules ameliorate ovariectomy-induced osteoporosis by the gut-bone-immune axis. It not only provides a molecular mechanism for elaborating Shengu granules to treat PMOP, but also provides a pharmaceutical candidate for PMOP treatment.

## Data availability statement

The original contributions presented in the study are included in the article/Supplementary material. Further inquiries can be directed to the corresponding authors.

## Ethics statement

The animal study was approved by Guangdong Provincial Second Hospital of Traditional Chinese Medicine. The study was conducted in accordance with the local legislation and institutional requirements.

## Author contributions

XC: Data curation, Investigation, Methodology, Software, Visualization, Writing – original draft. WL: Formal analysis, Investigation, Methodology, Visualization. JZ: Software, Visualization, Writing – original draft. YD: Investigation, Software, Visualization. JQ: Data curation, Investigation. BZ: Data curation, Investigation. DW: Data curation, Investigation. JL: Supervision, Validation, Visualization, Writing – review & editing. ZL: Methodology, Project administration, Resources, Supervision, Validation, Visualization, Writing – review & editing.
