# Supplementary material for: Shengu granules ameliorate ovariectomy-induced osteoporosis by the gut-bone-immune axis
Source: Front Microbiol. 2024 Mar 8;15:1320500. doi: 10.3389/fmicb.2024.1320500 (PMC10959285; doi:10.3389/fmicb.2024.1320500)
Supplement: Supplementary file 1 [file Data_Sheet_1.docx]

Supplementary material

**Supplementary information for materials and methods**

**Sequencing of rats’s feces**

**1. Kits and Instruments**

**1.1. Kits**

| **Kits Name** | **Producers** | **Cat. No.** |
| --- | --- | --- |
| MagPure Soil DNA LQ Kit | Magen | D6356-02 |
| Qubit dsDNA Assay Kit | Life Technologies | Q32854 |
| Tks Gflex DNA Polymerase | Takara | R060B |

**1.2. Instrument**

| **Instrument Name** | **Producers** | **Cat. No.** |
| --- | --- | --- |
| Centrifuge | Eppendorf | Centrifuge 5418 |
| PCR | Bio-rad | 580BR10905 |
| DNA Electrophoresis Cell | Tanon | HE-120 |
| Gel Imaging System | Tanon | 2500 |
| Pipette | Eppendorf | - |
| Bioanalyzer | Aglient | 2100 |
| NanoDrop | Thermo Fisher | 2100 |
| Tips, Centrifuge Tube | Axygen | - |

**2. Expeimental Proced**

**2.1. DNA extraction**

Total genomic DNA was extracted using DNA Extraction Kit following the manufacturer’s instructions. Concentration of DNA was verified with NanoDrop and agarose gel. The genome DNA was used as template for PCR amplification with the barcoded primers and Tks Gflex DNA Polymerase (Takara). For bacterial diversity analysis, V3-V4 (or V4-V5) variable regions of 16S rRNA genes was amplified with universal primers 343 F and 798 R (or 515F and 907R for V4-V5 regions).

2.2. Library Construction

Amplicon quality was visualized using gel electrophoresis, purified with AMPure XP beads (Agencourt), and amplified for another round of PCR. After purified with the AMPure XP beads again, the final amplicon was quantified using Qubit dsDNA assay kit. Equal amounts of purified amplicon were pooled for subsequent sequencing.

2.2.1. First Round PCR Reaction

1. Add the reaction system to the PCR tubes.

| **Name** | **Volume** |
| --- | --- |
| 2×Gflex PCR Buffer | 15 μl |
| 5 pmol/μl primer F | 1 μl |
| 5 pmol/μl primer R | 1 μl |
| Template DNA | ≥ 1 μl (50 ng) |
| Tks Gflex DNA Polymerase (1.25U/μl) | 0.6 μl |
| H_2_O | Add to 30 μl |
| Total | 30 μl |

1. Set up the PCR instrument according to the following procedure

| **Temperature** | **Time** | **Cycle Number** |
| --- | --- | --- |
| 94 °C | 5 min | 1 |
| 94 °C | 30 S | 26 |
| 56 °C | 30 S |  |
| 72 °C | 20 S |  |
| 72 °C | 5 min | 1 |
| 4 °C | hold | - |

2.2.2. Second Round PCR Reaction

a. Add the reaction system to the PCR tubes

| **Name** | **Volume** |
| --- | --- |
| 2×Gflex PCR Buffer | 15 μl |
| Tks Gflex DNA Polymerase (1.25U/μl) | 0.6 μl |
| Adapter I5 | 1 μl |
| Adapter I7 | 1 μl |
| First round product | Take 50 ng |
| H_2_O | Supplemented to 30 μl |
| Total | 30 μl |

1. Set up the PCR instrument according to the following procedure

| **Temperature** | **Time** | **Cycle Number** |
| --- | --- | --- |
| 94 °C | 5 min | 1 |
| 94 °C | 30 S | 7 |
| 56 °C | 30 S |  |
| 72 °C | 20 S |  |
| 72 °C | 5 min | 1 |
| 4 °C | hold | - |

**2.3. Magnetic Beads Purification**

a. Add 20 μl fully mixed AMPure XP beads (beads: product = 0.8: 1) to the U-shaped plate, and then add the PCR products. Blow them slowly with the pipette gun for 10 times, mix well, and store at room temperature for 5 min.

b. Place on the magnetic rack for 5 min to clear the supernatant and discard the upper clear.

c. Add 200 μl of freshly 80% ethanol at room temperature for 30 s and discard the supernatant.

d. Repeat the previous step and wash twice.

e. Dry the beads at room temperature until there is no droplet residue (check whether there are droplets at the bottom of the tube with a 10μl pipette), and there is no reflection on the surface of the beads, remove the plate from the magnetic frame.

f. Add 25 μl H2O for elution, blow 10 times, and mix thoroughly at room temperature for 2 min.

g. Place the supernatant on the magnetic rack for 5 min until the supernatant is transparent and transfer 20 μl supernatant to a new PCR tube.

**3. Detection**

a. 5μl of purified secondary products were detected by agarose gel electrophoresis to detect whether there were bands and whether the bands were single or not.

b. Take 1 μl of purified secondary product and detect the concentration by Qubit.

| **Sequence Type** | **Primer** | **Primer Sequence** |
| --- | --- | --- |
| Bacteria 16S rRNA | 343F | 5'- TACGGRAGGCAGCAG -3' |
| V3-V4 region | 798R | 5'- AGGGTATCTAATCCT-3' |

**SCFAs analysis**

**1. Sample Collection and preservation**

Feces were frozen immediately after collection and stored at − 80 ° C until further analysis. The target metabolites were Acetic Acid, Propionic acid, Butyric Acid, Pentanoic acid, Hexanoic Acid, and Isobutyric acid acid) and Isovaleric acid.

**2. Sample pretreatment**

(1) Take an appropriate amount of sample, add 300 μL 50% acetone-water solution (v/v, containing internal standard [2H9]-Pentanoic acid, [2H11]-Hexanoic Acid) (precooled at 4℃);

(2) The samples were ground for 3 min (the sample tray was precooled at -20℃) and extracted by ultrasonic for 10 min in an ice water bath.

(3) Centrifugation (10 min, 4℃, 12000 rpm), and the supernatant was diluted 5 times with 50% acetonitrile-water solution (V/V);

(4) 80 μL of diluted supernatant was transferred to injection vials (bandwidth liner).

**3. Sample derivatization**

(1) Derivatization: 40 μL of 200mM 3-NPH (50% acetonitrile-aqueous solution, v/v) and 40 μL of 120mM EDC-6% pyridine (50% acetonitrile-aqueous solution, v/v) were added to the injection glass bottle containing the extract, and the reaction was carried out at 40 ° C for 30min.

(2) After cooling on ice for 1min, 160μL of the supernatant was sucked by a syringe, filtered by a 0.22μm organic phase pinhole filter, transferred to a brown injection bottle, and stored at -80℃ until analysis.

**4. Derivatization of standards**

(1) Derivatization: Add 40 μL of 200mM 3-NPH (50% acetonitrile-aqueous solution, V/V) and 40 μL of 120mM EDC-6% pyridine (50% acetonitrile-aqueous solution, V/V) to 80μL of standard into the injection glass bottle, and react at 40℃ for 30min.

(2) After cooling on ice for 1min, 160μL of the supernatant was sucked by a syringe, filtered through a 0.22μm organic phase pinhole filter, transferred to a brown injection bottle, and stored at -80℃ until analysis.

Remarks: (1) All extraction reagents were precooled at -20 ° C before use. (2) Quality control samples (QC) were prepared by mixing the extracts of all samples in equal volume.

**5. Chromatography mass spectrometry method**

In this experiment, UPLC-ESI-MS/MS analysis method was used to qualitatively and quantitatively detect the target metabolites. The specific analysis conditions and methods were as follows:

(1) Chromatographic conditions: injection volume: 2 μL; Flow rate: 0.4mL/min; Mobile phase: A(0.1% formic acid-aqueous solution), B(acetonitrile/methanol =2:1); Gradient Elution Procedures: 0 min A/B (80:20, V/V), 2 min A/B (80:20, V/V), 8min A/B (60:40, V/V), 8.1min A/B (5:95, V/V), 9.5 min A/B (5:95, V/V), 9.6 min A/B (80:20, V/V), 10 min A/B (80:20, V/V).

(2) Mass spectrum conditions: curtain gas: 35(psi); collision-activated dissociation (CAD) parameters: medium; Positive ion spray voltage: 5500V; Negative ion spray voltage: -4500V; Ion source temperature: 450℃ column temperature: 40℃; Spray gas (Gas1):50(psi); Auxiliary heating gas (Gas2) : 60(psi).

**6. Qualitative and quantitative principles**

Metabolites were quantified using the multiple reaction detection (MRM) mode of triple quadrupole mass spectrometry. In MRM mode, the quadrupole first screens the precursor ions (parent ions) of the target substance, and excludes the ions corresponding to other molecular weight substances to preliminarily exclude interference. After the precursor ion is induced by the collision chamber, it breaks to form many fragment ions. The fragment ions are filtered through the triple quadrupole to select a required characteristic fragment ion, which eliminates the interference of non-target ions and makes the quantification more accurate and reproducible. The mass spectrometry data of different samples were obtained, and the peak area of all chromatographic peaks was integrated, and the chromatographic peaks of the same substance in different samples were integrated and corrected.

1. **SCFAs analysis Supplementary tables**

**Standard and reagent information sheet**

| **Reagent** | **Specification** | **Producers** | **Cat.NO.** |
| --- | --- | --- | --- |
| Methanol | HPLC | Fisher | A452-4 |
| Water | HPLC | WAHAHA | / |
| Formic Acid | HPLC | Fisher | A452-4 |
| Acetonitrile | HPLC | Fisher | A998-4 |
| (3-Nitrophenyl)hydrazine | HPLC | Sigma | N21804-25g |
| N- (3-dimethylaminopropyl) -N '-ethylcarbodiimide hydrochloride | HPLC | Aladdin | E106172-100g |

**Instrument Information sheet**

| **Instrument Name** | **Producers** | **Cat.NO.** |
| --- | --- | --- |
| Grinding Mill | Wonbio | Wonbio-E |
| Ultrasonic Cleaners | FUYANG | F-060SD |
| Water Bath | Shanghai Kexin | HMTD203 |
| Vortex Oscillator |  | TYXH-I |
| Centrifuge | Bioridge | TGL-16MS |
| Mass Spectrograph | AB Sciex | AB Sciex Qtrap 5500 |
| Liquid Chromatograph | SHIMADZU | Nexera UHPLC LC-30A |
| Chromatographic Column | Waters | ACQUITY UPLC BEH C18 (100*2.1 mm,1.7 μm) |

**Histopathological scores Supplementary tables**

**Table 1 Histopathological scores**

| **Score** | **Inflammation** | **Crypt Damage** | **Ulceration** | **Edema** |
| --- | --- | --- | --- | --- |
| 0 | No infiltrate | None | None | None |
| 1 | Occasional cell limited to submucosa | Some crypt damage, spaces between crypts | Small, focal ulcers | Present |
| 2 | Significant presence of inflammatory cells in submucosa, limited to focal areas | Larger spaces between crypts, loss of goblet cells, some shortening of crypts | Frequent small ulcers |  |
| 3 | Infiltrate present in both submucosa and lamina propria, limited to focal areas | Large areas without crypts, surrounded by normal crypts | Large areas lacking surface epithelium |  |
| 4 | Large amount of infiltrate in submucosa, lamina propria and surrounding blood vessels, covering large areas of mucosa | No crypts |  |  |
| 5 | Transmural inflammation (mucosa to muscularis) |  |  |  |
